# Neural mass modeling reveals that hyperexcitability underpins slow‐wave sleep changes in children with epilepsy

**DOI:** 10.1111/epi.18293

**Published:** 2025-02-07

**Authors:** Dominic M. Dunstan, Samantha Y. S. Chan, Marc Goodfellow

**Affiliations:** ^1^ Department of Mathematics and Statistics University of Exeter Exeter UK; ^2^ Living Systems Institute University of Exeter Exeter UK; ^3^ St George's Hospital London UK; ^4^ University College London Great Ormond Street Institute of Child Health London UK; ^5^ St George's University of London London UK

**Keywords:** children, EEG, epilepsy, neural mass model, sleep

## Abstract

**Objective:**

The relationship between sleep and epilepsy is important but imperfectly understood. We sought to understand the mechanisms that explain the differences in sleep homeostasis observed in children with epilepsy.

**Methods:**

We used a neural mass model to replicate sleep electroencephalography (EEG) recorded from 15 children with focal lesional epilepsies and 16 healthy age‐matched controls. Different parameter sets were recovered in the model for each subject.

**Results:**

The model revealed that sleep EEG differences are driven by enhanced firing rates in the neuronal populations of patients, which arise predominantly due to enhanced excitatory synaptic currents. These differences were more marked in patients who had seizures within 72 h after the sleep recording. Furthermore, model parameters inferred from patients resided closer to model parameters inferred from a typical seizure rhythm.

**Significance:**

These results demonstrate that brain mechanisms relating to epilepsy manifest in the interictal EEG in slow‐wave sleep, and that EEG recorded from patients can be mapped to synaptic deficits that may explain their predisposition to seizures. Neural mass models inferred from sleep EEG data have the potential to generate new biomarkers to predict seizure occurrence and inform treatment decisions.

**Key points**

- The mechanisms that differentiate children with epilepsy from controls during slow‐wave sleep can be understood using a mathematical model.
- The observed spectral power shifts in patients are predominately explained by greater excitatory synaptic currents.
- These differences in currents place patients closer to seizure rhythms in the model.
- Ultimately, this framework could help foster the development of biomarkers to guide intervention in epilepsy.

## 1 INTRODUCTION

The electroencephalogram (EEG) in deep sleep is dominated by slow waves of high amplitude, generated by widespread neurons alternating in synchrony between depolarized and hyperpolarized states.^1^ This slow‐wave activity (SWA) correlates closely with sleep need, building up with time spent awake and dissipating with sleep.^2^ It has been proposed that the decrease in global SWA across the night reflects the process of synaptic renormalization,^3^ a homeostatic mechanism that regulates cortical excitability and facilitates neural plasticity.^4^ Furthermore, it is theorized that the disruption of sleep homeostasis may be the basis of sleep‐related epilepsies with childhood onset, ranging from self‐limited childhood focal epilepsies to mesial temporal lobe epilepsy.^5^ In support of this notion, Eriksson et al.^6^ recently observed that the dynamics of SWA in children with focal lesional epilepsies differed from age‐matched healthy controls, with the epilepsy patients producing less SWA during the first hour of the sleep recording. Moreover, these differences were found to be exacerbated in patients with a higher propensity to seizures.^6^

Understanding the physiological mechanisms that contribute to differences observed in the EEG in disease is a challenging problem.^7^ Computational models can help; if we can match the simulations generated by models to data recorded from human subjects, we can then examine which components, parameters, or settings of the model were crucial to allow it to generate the data of interest. Several models of EEG have been developed and have been shown to generate dynamics similar to a variety of resting and pathological states.^8^ In particular, neural mass models parsimoniously capture synaptic interactions between populations of excitatory and inhibitory neurons.^8^ This allows for EEG brain rhythms to be understood in terms of synaptic dynamics, connectivity, and firing rates at the level of brain tissue.^8^, ^9^, ^10^

An important technical challenge is how to match model dynamics to data. Various methods are available for this, including routines within dynamic causal modeling.^11^ Such approaches often use linear models,^12^ combined with prior beliefs on parameter values. The latter are difficult to ascertain for neural mass models, and the former does not capture the nonlinear dynamics of the brain and, in particular, the nonlinear mechanisms of epileptiform EEG.^13^ Recently, we have demonstrated the promise of multiobjective optimization as a global nonlinear approach that can be used to model resting and pathological EEG.^14^ This method enables the efficient search of parameter space to identify parameter values in the model that, when simulated, produce an output that recapitulates desired features of the data. This process can be applied to data from patients and healthy controls to understand the mechanisms (neural mass model parameters) that are responsible for changes observed in the EEG. Here, we apply this approach to decipher the mechanisms in the sleeping cortex^15^ that contribute to the differences in SWA observed in patients with epilepsy. We then explore perturbations to the model that could potentially rectify the different dynamics observed in epilepsy. Finally, having identified mechanisms underpinning differences in resting EEG, we link these mechanisms to the generation of seizures. Here, we do this by quantifying the proximity (in terms of similarity of recovered parameter values) of resting dynamics to dynamics of an archetypal seizure rhythm.

## 2 MATERIALS AND METHODS

### 2.1 Participants and study design

Participants consisted of 15 children with drug‐resistant focal epilepsy of structural (or presumed structural) etiology, and 16 age‐ and sex‐matched typically developing controls. Patients were recruited prospectively from the EEG video telemetry unit at Great Ormond Street Hospital as described previously.^6^, ^16^ Children were aged between 6 and 16 years, and EEG was recorded continuously during planned four‐night hospital admissions. Control participants were recruited by advertisements directed at staff working at the UK charity Young Epilepsy. Controls attended the EEG department of Young Epilepsy to be set up for a single‐night ambulatory sleep study. Compared to the previous cohort,^6^ four patients and two controls were excluded due to artifacts in the recordings. Group differences in demographic and clinical data were examined using independent samples *t*‐tests for continuous variables and chi‐squared tests for categorical variables.

### 2.2 EEG data acquisition and preprocessing

EEG polysomnography acquisition, visual sleep scoring, and the visual quantification and marking of seizures have been described in previous work.^16^ EEG data were recorded with the Xltek Trex system (Natus Medical Incorporated) at 512 Hz using a 10–10 montage (Fz, Cz, Pz, Fp1, Fp2, F3, F4, F7, F8, F9, F10, C3, C4, C5, C6, T5, T6, T7, T8, T9, T10, P3, P4, P9, P10, O1, O2) in patients and at 256 Hz using an eight‐electrode montage (F3, F4, C3, C4, O1, O2, A1, A2) in controls. Recordings were downsampled to 128 Hz in Natus Sleepworks before exporting as an .edf file for offline analysis. The full EEG recording (all available channels) was reviewed visually for artifacts in EDFbrowser (version 1.88, https://www.teuniz.net/edfbrowser/), and channels marred by artifacts were excluded. No artifact removal was performed.

To identify early night SWA, the recordings were viewed on a whole night timescale with color density spectral array to identify segments with high amounts of 1–4 Hz with or preceded by high amounts of 10–12 Hz occurring within the first 2 h of sleep. The identified segments were reviewed again at a 10‐s per‐page scale with all channels for visual identification of SWA before cropping. The mean activity recorded from the frontal electrodes (F3, F4) was used for further analysis.

To estimate the subjects' power spectral density from the sleep EEG data, we split eligible segments into 30‐s epochs, with each epoch filtered via a 4th order Butterworth filter between .3 and 10 Hz. Power spectra were estimated from each epoch across .6–10 Hz, with .025‐Hz resolution. This was performed using Welch's method with 50% overlap. Spectra across epochs were then averaged to obtain a mean waveform for each subject. The power spectra were then normalized by dividing by the total area under the curve within .6–10 Hz. This normalization ensured the relative prominence of each frequency component was compared across subjects.^17^

### 2.3 Parent‐rated sleep disturbance

Parents were asked to rate the frequency of various sleep behaviors as they would occur in a typical week using the Children's Sleep Habits Questionnaire.^18^

### 2.4 Modeling framework

A neural mass model was used to simulate the temporal dynamics of mean membrane potentials and firing rates in a cortical region.^19^ Specifically, we used a conductance‐based neural mass model developed to model non‐rapid eye movement sleep EEG by Weigenand et al.^15^ Here, neurons are grouped into interacting excitatory and inhibitory populations, and excitatory, inhibitory, leak, and sodium‐dependent potassium (KNa) synaptic currents (at the level of the neural mass) are tracked over time. We note that excitatory synaptic currents exhibit efferent depolarization (labeled as α‐amino‐3‐hydroxy‐5‐methyl‐4‐isoxazolepropionic acid [AMPA] in Weigenand et al.^15^) and inhibitory synaptic currents exhibit efferent hyperpolarization (labeled as γ‐aminobutyric acid [GABA] in Weigenand et al.^15^). Furthermore, KNa synaptic currents have been suggested as a mechanism for slow oscillations.^15^, ^20^ We used MATLAB^21^ to numerically solve the model. Additional details of the model, including model equations, can be found in Appendix S1.

The model comprises 32 parameters. These parameters describe the mean synaptic interactions between excitatory and inhibitory neuronal populations. These facilitate a mechanistic interpretation of the neuronal activity analogous to an EEG recording, without explicitly modeling the activity generated by single cells.^8^ However, parameters at this scale are difficult to constrain, and simulating the model with different parameter values results in different dynamics. Therefore, we implemented a previously developed multiobjective genetic algorithm^14^ to search model parameter space for combinations of parameters that yield model simulations with properties similar to those of the EEG. Table S1 defines the model parameters, gives a brief description of their interpretation, and defines the lower and upper parameter bounds that form the search space.

**FIGURE 1:**
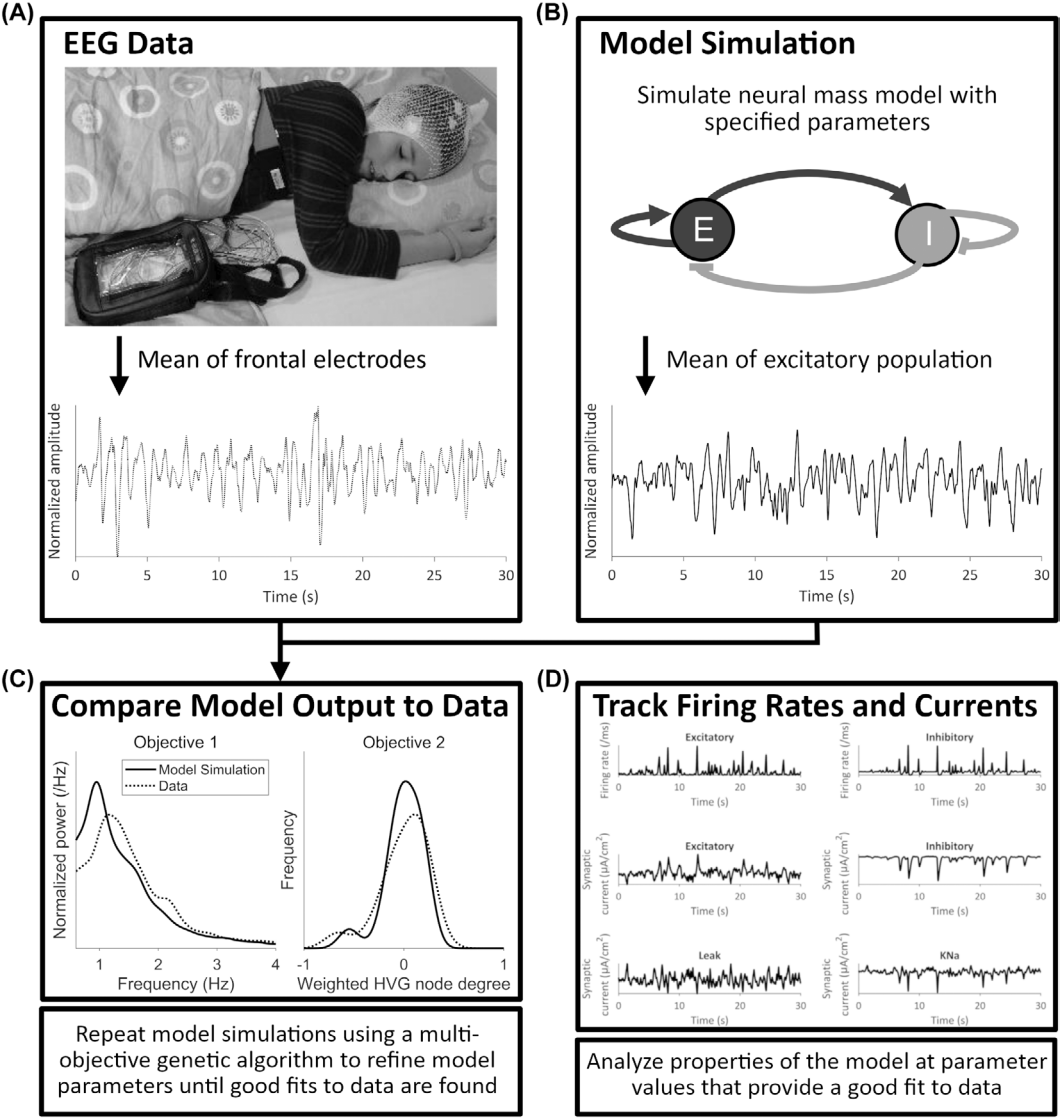
Illustration of the process of comparing model output with data to obtain parameters that explain the electroencephalogram (EEG). (A) Sample patient EEG recording during sleep. The means from the F3 and F4 electrodes are used in the analysis. (B) Schematic of the neural mass model used, consisting of interacting excitatory (E) and inhibitory (I) populations of neurons. The output of the model is a 30‐s time series of simulated EEG. (C) Model output is compared to the data by defining objectives to recapitulate in the simulations. These objectives are based on the normalized power (objective 1) and the weighted horizontal visibility graph (HVG; objective 2). Model parameters are adjusted to find simulations that aim to minimize the objectives (see Section 2). (D) Several properties of the neural mass model are analyzed, including firing rates and synaptic currents. KNa, sodium‐dependent potassium.

To quantitatively compare model output with data, objectives that describe the difference between the model and data were defined, and the algorithm was used to recover solutions that minimized these objectives. Following our previous work,^14^ we defined two objectives: (1) the difference in normalized power and (2) the difference in node degree of the weighted horizontal visibility graph (HVG). The latter objective maps the EEG time series to a network and has been shown to distinguish between stochastic processes and nonlinear dynamics, including epileptiform rhythms.^14^, ^22^, ^23^ Figure S3 shows an example simulation recovered using the single objective optimization approach. The addition of the weighted HVG objective more accurately refines plausible simulations recovered and improves model parameter identifiability, as discussed in Appendix S2. The optimization was repeated 100 times for each subject to obtain a distribution of parameters that could describe the subject's EEG data. These repeats in the optimization allow for the parameter unidentifiability to be accounted for. Figure 1 provides an overview of how parameters were recovered by comparing the model output to the recorded EEG. For further details regarding the definition of objectives, and information on how the model output is aligned to data, see Appendix S2.

### 2.5 Comparison of resting activity to seizure activity

We additionally optimized the model to generate spike–wave discharges (SWDs). We note that this waveform consisted of a single 2.5‐s segment of data. A 4th order high‐pass Butterworth filter was applied to this time series to remove low‐frequency artifacts. We used the same optimization algorithm and objectives to recover the model parameters that simulate the SWD as we did for the control and patient SWA resting data. Optimizing to this rhythm therefore enabled us to compare (in silico) how the mechanisms of a resting state relate to mechanisms of a seizure state by contrasting model parameters (or emergent properties) that were inferred from simulating each of the states. Further information on the optimization process used is provided in Appendix S2. Although some other approaches have used thalamocortical models to simulate SWDs,^24^ cortical neural mass models and simpler neural mass models with slow–fast dynamics have also demonstrated SWDs, suggesting different mechanisms in principle (e.g., see Goodfellow et al.,^9^ Wang et al.^25^). In this work, we use a cortical model, as it enables us to directly relate parameters from the resting state to the seizure state and allows for a more computationally efficient simulation of the SWD.

We specifically used the SWD in this work as it is the archetypal waveform seen on surface EEG during a generalized seizure^26^ and is thought to have a cortical origin.^27^ SWDs are activated across a spectrum of common childhood epilepsies, such as self‐limited epilepsy with centrotemporal spikes^28^ and various genetic generalized epilepsies.^29^ The SWD also bears a special relationship with sleep slow waves, particularly in the pediatric age group. SWDs occur more frequently during sleep across the epilepsies and, in the spectrum of epileptic encephalopathies with continuous spike–wave during sleep, may sometimes replace slow waves altogether.^30^ Our sample falls both within the age range and within the range of underlying etiologies in which this phenomenon may be seen. In animal models, SWDs have been observed to develop from sleeplike slow rhythms.^31^ Furthermore, patients with epilepsy can exhibit a wide range of seizure onset patterns, but often these seizures progress electrographically to SWDs. The relative temporal stability of SWD dynamics facilitates model fitting, and the SWD has been recreated in a variety of cortical and thalamocortical neural mass models.^9^, ^14^, ^24^, ^25^ Therefore, in this work, we chose to focus on the SWD as the generic rhythm to simulate seizures in the model.

### 2.6 In silico prediction for intervention

To understand how intervention may alter the dynamics observed on the EEG, we investigated the sensitivity of the power spectrum to changes in model parameters. In particular, we recorded the simulated power spectrum after individually adjusting the parameters governing each synaptic channel conductance (excitatory, inhibitory, leak, and KNa). We focused on perturbing the conductance parameters in the model because these parameters most directly relate to a key mechanistic target of antiseizure medications (e.g., see Sills^32^). This includes benzodiazepines, which have been shown to increase the opening probability of chloride channels activated by GABA,^33^ as well as potentially increasing the conductance of GABA_A_ channels directly.^34^ Furthermore, recent work has shown perampanel can block AMPA‐mediated synaptic conductances.^35^ Hence, to simulate the effects of treatment at the scale of the model, we decreased the excitatory and leak conductances and increased the inhibitory and KNa conductances in the model parameters derived from patient data.

### 2.7 Statistical testing

Throughout this work, unless stated otherwise, we use a nonparametric Mann–Whitney *U*‐test to compare among groups. In cases where multiple comparisons were made (e.g., four types of synaptic currents were compared), we state a Bonferroni‐corrected *p*‐value to compensate for the multiple comparisons problem.

## 3 RESULTS

### 3.1 Clinical characteristics of participants

Participant demographics and patient clinical characteristics are summarized in Table 1. In particular, no significant differences in age or sex were recorded between the cohorts. Further information on the epilepsy characteristics of patients is provided in Table S2.

**TABLE 1:** Population demographics.

Characteristic	Cohort		*p*
	Patients, *n* = 15	Controls, *n* = 16	
Male (female)	11 (4)	7 (9)	.0953
Age, years, mean (SD)	11.4 (2.97)	9.86 (2.72)	.132
CSHQ, mean (SD)	49.9 (9.14)	37.5 (3.62)	<.001
Full‐scale IQ, mean (SD)	85.6 (11.0)	111 (9.66)	<.001
Epilepsy characteristics			
Age at onset of seizures, years, mean (SD)	5.21 (3.92)		
Duration of epilepsy, years, mean (SD)	6.23 (2.57)		
MRI positive (negative)	10 (5)		
Nocturnal seizures			
Every night	3		
Sometimes	8		
Never	4		
Reported seizure frequency			
Daily	3		
Weekly	6		
Monthly	3		
<1 per month	3		
Seizure focus			
Frontal	3		
Temporal	3		
Frontotemporal	4		
Parietal	1		
Occipital	1		
Undetermined	3		
Number of antiseizure medications			
None	1		
One	8		
Two	4		
Three	2		
Seizure(s) during admission, yes (no)	7 (8)		

*Note*: Probability values were generated from independent samples *t*‐tests for continuous variables and chi‐squared tests for categorical variables.

Abbreviations: CSHQ, Children's Sleep Habits Questionnaire; IQ, intelligence quotient; MRI, magnetic resonance imaging.

### 3.2 Normalized power spectra in controls and patients

**FIGURE 2:**
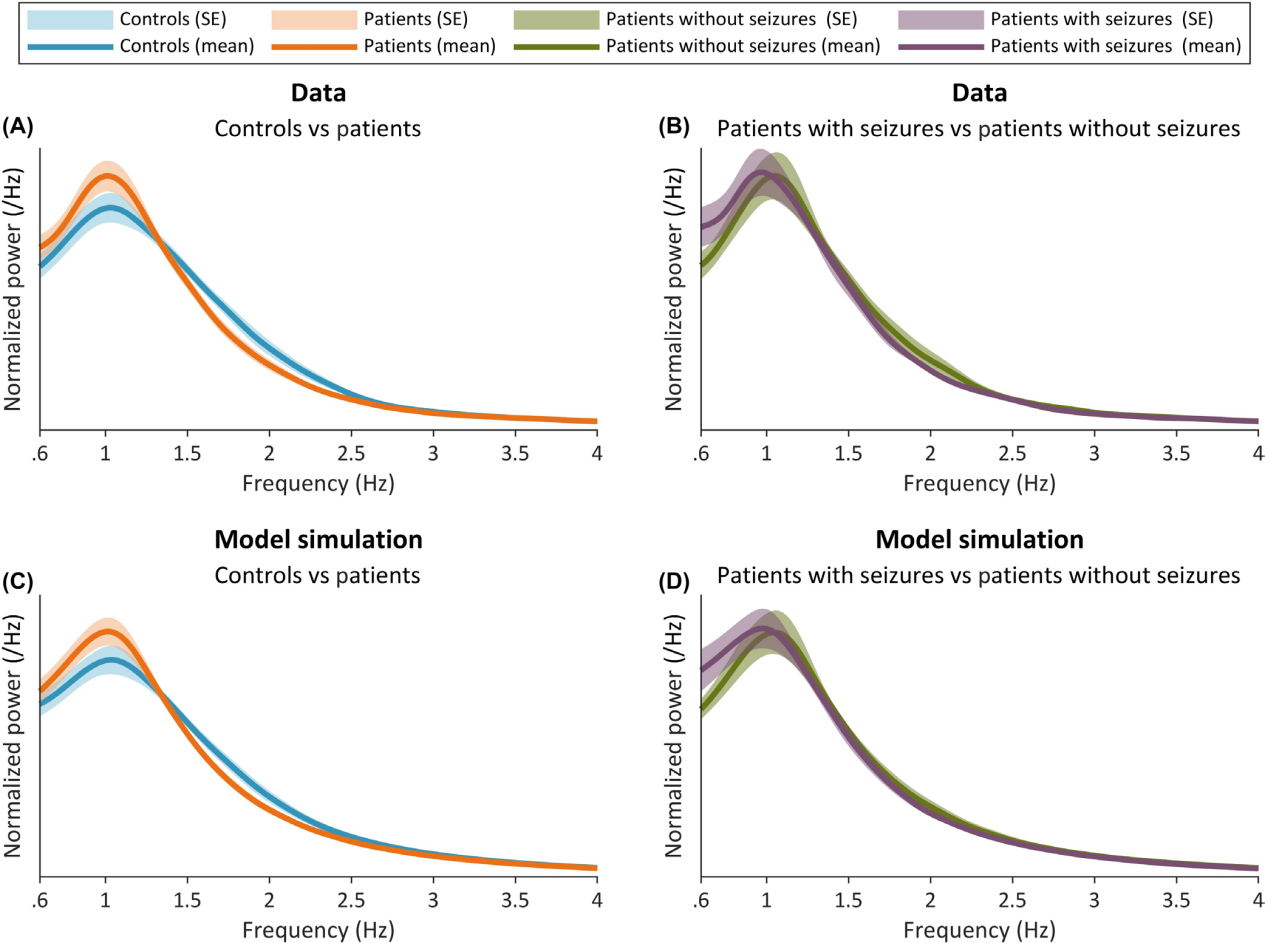
(A) Normalized power from control and patient data (mean and SE across subjects). (B) Normalized power across patients after splitting patients who had a seizure during their admission (patients with seizures) and patients who did not have a seizure during their admission (patients without seizures). (C, D) Same as panels A and B, but for model simulations after optimizing the dynamics to data. Differences in the mean and SE normalized power between groups were recapitulated in the model simulations.

Spectral analysis of the normalized EEG revealed that patients had less relative power in the higher delta range (1.5–4 Hz) than controls (Figure 2A) and more relative power in the lower delta range (.6–1.5 Hz) than controls (Figure 2A). Patients who had a seizure during their hospital admission (referred to herein as "patients with seizures") had even less power in this range than those who did not (referred to herein as "patients without seizures"; Figure 2B). The mean and SE normalized power from model simulations are shown in Figure 2C,D. It can be seen that the simulations qualitatively re‐create the normalized power of the data and the difference observed between each of the groups. Furthermore, for sample control and patient subjects, normalized power and time series from the model output are given in Appendix S4.

### 3.3 Comparison of model simulations recovered from controls and patients

**FIGURE 3:**
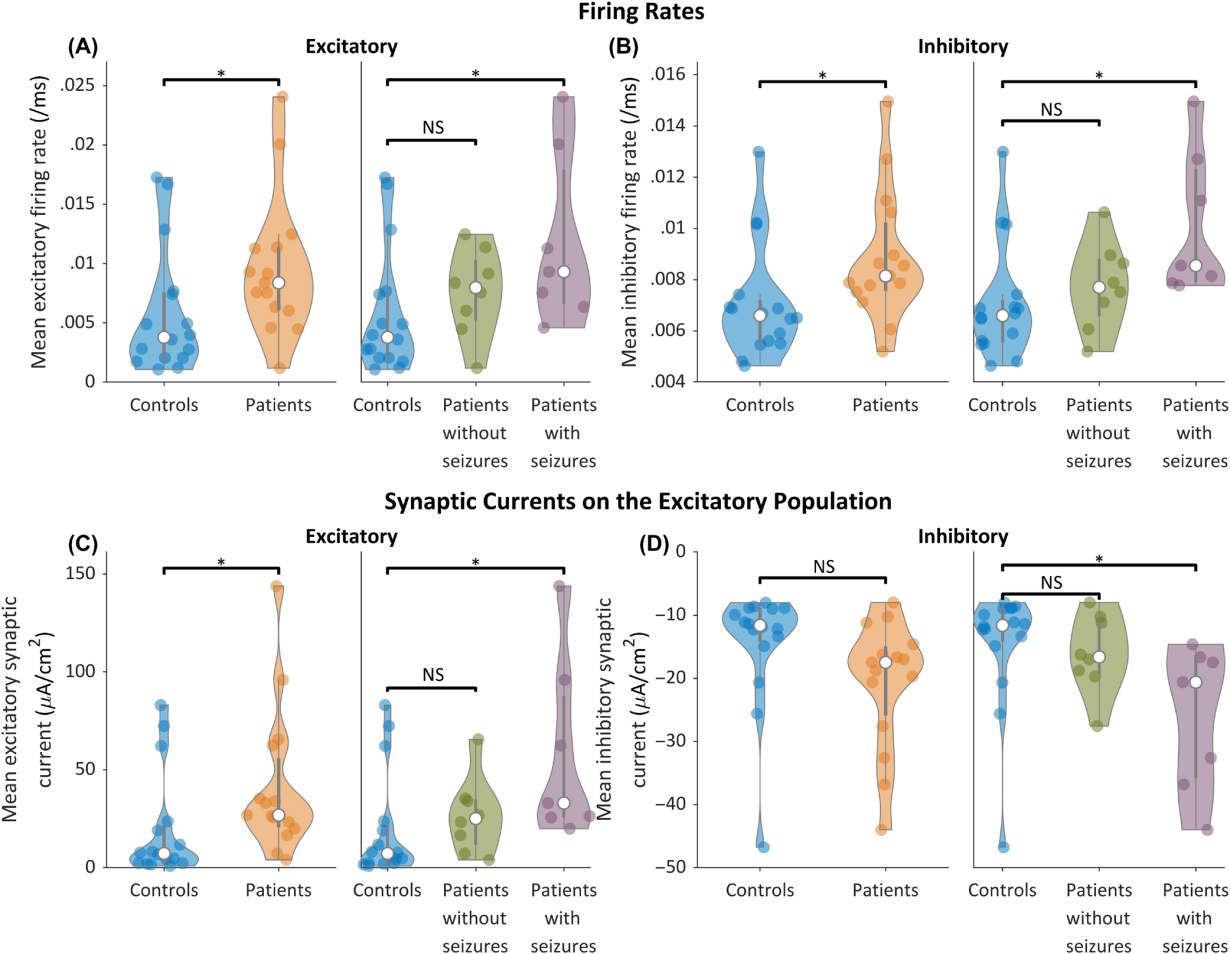
(A) Excitatory and (B) inhibitory mean firing rates obtained from model simulations. Patients had greater mean excitatory and inhibitory firing rates than controls, as did the subset of patients with seizures during their admission. The mean firing rate of patients without seizures during their admission was not significantly different from controls. (C) Excitatory and (D) inhibitory synaptic currents on the excitatory neuronal population, obtained from model simulations. In each case, each point on a violin plot gives the mean value of a subject. *p < .05 after Bonferroni correction, NS = not significant.

Parameter distributions recovered from optimizing to control and patient data are shown in Figure S6. This figure also ranks the Jansen–Shannon divergence between the control and patient distributions of each parameter. Significant differences were found (see Appendix S4 for information on statistical testing across distributions) in synaptic time scales (*γ*_*e*_ and *γ*_*i*_), excitatory connectivity to excitatory and inhibitory neuronal populations (*N*_*ee*_ and *N*_*ei*_), firing rate thresholds (*θ*_*e*_ and *θ*_*i*_) and the excitatory synaptic reversal potential (*E*_*e*_). Of these, parameter *θ*_*e*_ showed the largest difference between controls and patients, with patient values lower than controls. This difference results in a greater excitatory firing rate in patients than in controls for a given membrane potential. Combined, these parameters contribute to differences in the emergent properties of the membrane potential and excitability. To interpret the differences observed, we analyzed the mean firing rates and mean synaptic currents obtained from the model simulations that best fit the data (Figure 3). It can be seen that patients, and specifically patients who had seizures during their admission, had higher mean firing rates in the excitatory and inhibitory neuronal populations (Figure 3A,B). Patients had significantly larger excitatory synaptic currents onto the excitatory neuronal population than controls (Figure 3C). These differences were driven by the subset of patients who had a seizure during their admittance. Patients with seizures also displayed significantly larger (more negative) inhibitory synaptic currents onto excitatory neurons than controls (Figure 3D). We note that no significant differences were observed in the leak and KNa synaptic currents on the excitatory neuronal populations (Figure S7), nor any of the synaptic currents on the inhibitory neuronal populations (Figure S8).

### 3.4 In silico predictions for intervention

**FIGURE 4:**
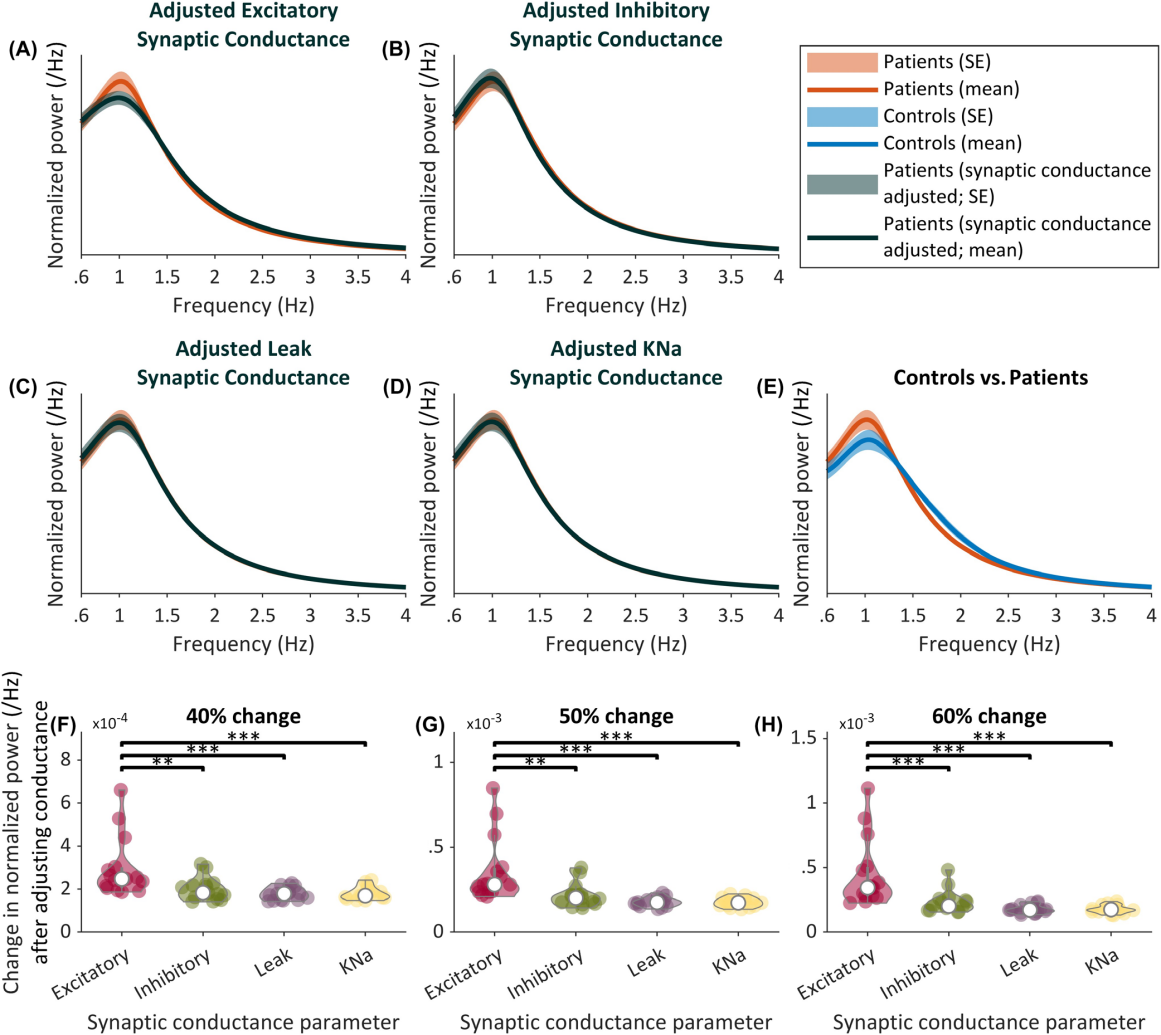
(A–D) Normalized power spectra of model simulations from all patients, along with the normalized power spectra obtained after adjusting the excitatory, inhibitory, leak, and sodium‐dependent potassium (KNa) synaptic conductances by 50% of their range, respectively. (E) Normalized power spectra of recorded control and patient electroencephalogram, for reference. (F–H) Change in normalized power spectra after adjusting the synaptic conductance parameter for 40%, 50%, and 60% of the specified parameter bounds (see Table S1), respectively. **p < .01 and ***p < .001.

To further understand the influence that each of the synaptic currents has on the power spectrum, we adjusted the conductance of each synapse and simulated the resulting power spectrum. We adjusted each conductance by 40%, 50%, or 60% of its bound (see Table S1), starting from the baseline recovered from simulating patient EEG. Note that the parameters governing the excitatory and leak synaptic conductance were reduced, whereas the parameters governing the inhibitory and KNa synaptic conductance were increased, in line with the trends observed in Figure 3. Also note that whereas the results of Figure 3 were specific to synaptic currents on the excitatory neuronal population, here we adjust the excitatory synaptic conductance in general. This allows for the simulation of the effects that drugs (such as antiseizure medications) have on the power spectrum. Figure 4 shows that reducing the excitatory synaptic conductance produced the largest change in the power spectrum. A 50% reduction in the excitatory conductance was sufficient to shift the mean patient power spectrum toward the mean control power spectrum, simulating a “normalization” of the sleep dynamics of patients (see Figure 4A,E). Moreover, the sensitivity of the power spectrum to changes in synaptic conductance was found to be significantly higher for the excitatory conductance than all of the other conductances in the model (Figure 4F–H).

### 3.5 Comparison of model configurations obtained between resting and seizure dynamics

**FIGURE 5:**
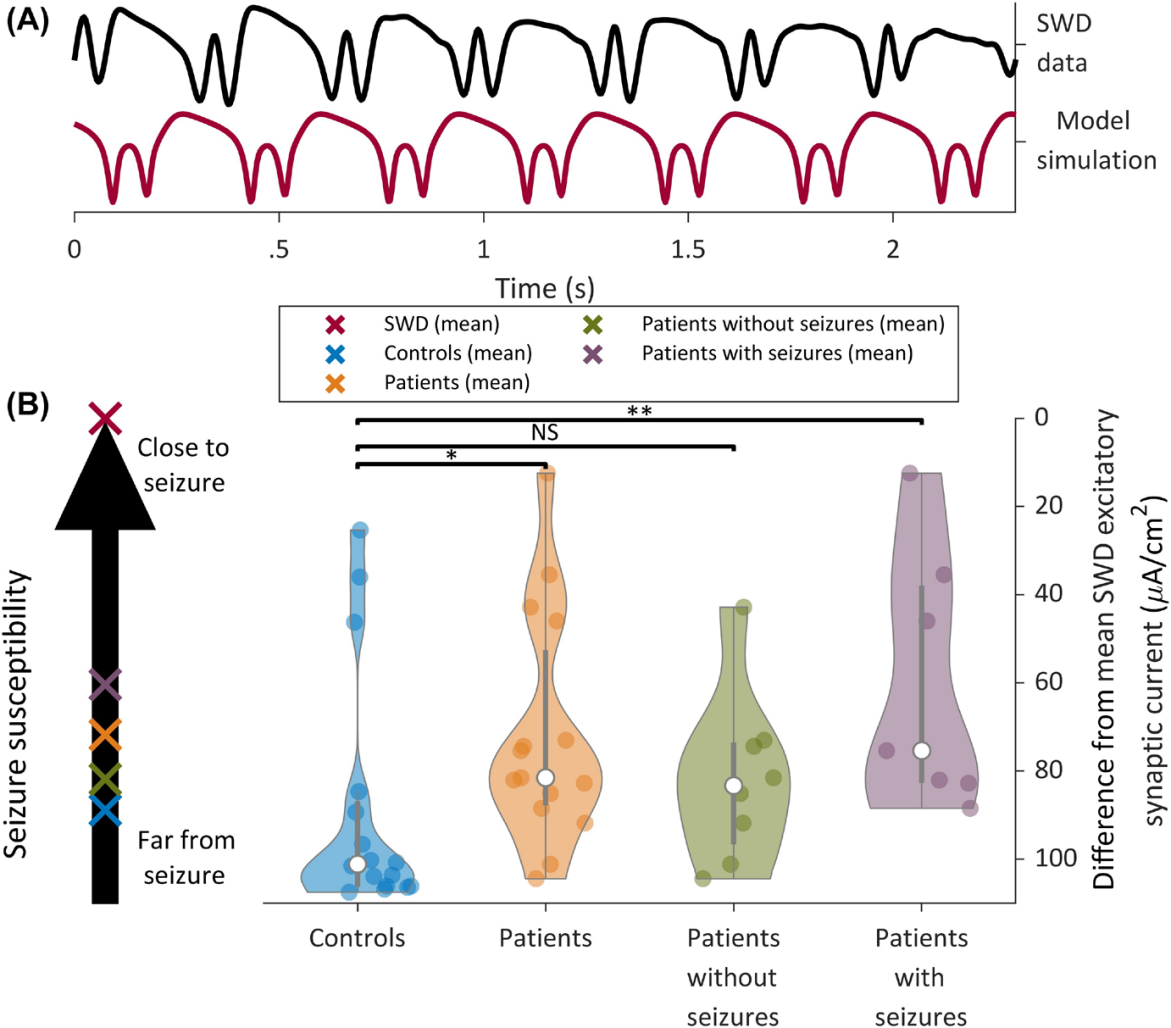
(A) Spike–wave discharge (SWD) data segment and example model simulation obtained after optimizing the model dynamics to SWD data. (B) Comparison of the absolute difference in excitatory synaptic current obtained from control and patient simulations (inferred from slow‐wave activity), compared to the excitatory synaptic current inferred from SWD simulations. This formed a continuum of seizure susceptibility, as shown on the left of panel B. Patients, and in particular the subset of patients who had a seizure during their admittance, had excitatory synaptic current more similar to the SWD than controls. *p < .05, **p < .01. NS, not significant.

Hitherto, we used the model to reveal hidden firing rates and synaptic dynamics underlying SWA. We found that alterations to the interactions between excitatory and inhibitory neuronal populations could be revealed from this resting EEG, which was free of overt pathological rhythms. We next sought to understand how these differences might be related to the generation of seizures, which is the defining characteristic of the patient group. To do this, we performed another in silico experiment, matching the model output to a prototypic seizure dynamic, the SWD. Figure 5A shows a 2.5‐s data segment during an SWD, along with a typical model simulation generated using parameters that were recovered by fitting the model to this rhythm. We note that as seen in the figure, the simulation from the cortical model used could accurately recapitulate the waveform of the SWD. We compared the mean excitatory synaptic currents inferred from the SWD data to those inferred from patient and control data (obtained from resting SWA). We observed that, compared to controls, patients, and in particular the subset of patients who had a seizure during their admission, had excitatory synaptic currents more similar in magnitude to the mean excitatory synaptic currents recovered from SWD simulations (Figure 5B). This implies that smaller changes to excitatory synaptic currents would cause patients to generate SWDs, compared to controls. Hence, the model parameters inferred only from SWA dynamics revealed a hidden ictogenicity, as well as a mechanistic explanation (enhanced excitatory synaptic currents and excitability).

## 4 DISCUSSION

We have used a neural mass model to recapitulate the EEG during deep sleep in children with epilepsy and age‐matched healthy controls. These models encapsulate essential features of the system of interest and, by optimizing their parameters to data, allow for inferences to be made about the mechanisms that are altered in disease.

In the cohort studied, optimizing the model parameters for each subject revealed that the differences observed in sleep homeostasis^6^ at the group level were explained by hyperexcitability in patients, driven predominantly by greater firing rates and excitatory synaptic currents. By comparing the excitatory synaptic current obtained from the resting sleep data with the excitatory synaptic current obtained from simulating an SWD, we were additionally able to show that patients were closer to a seizure state than controls. Our findings demonstrate that hypotheses regarding the mechanisms that contribute to the propensity of the brain to generate seizures, which is hidden from a visual analysis of the EEG, can be revealed by mathematical modeling. Crucially, by modifying the parameters governing ion channel conductances in the model, we were also able to simulate the effects that clinical intervention might have.

Experiments have indicated that cortical excitation/inhibition balance is not fixed, but rather changes across different brain states and across the sleep–wake cycle.^36^, ^37^ In particular, it has been observed that slow‐wave sleep has the largest excitation/inhibition ratio across all the sleep stages.^36^, ^37^ In line with this, we have found that slow‐wave sleep EEG can reveal synaptic deficits associated with increased excitation in epilepsy, a condition thought to arise from an enhanced excitation/inhibition ratio. In our study, we additionally showed that these differences in excitation placed patients closer to seizures than controls, thereby demonstrating that a smaller adjustment to the patient model could lead them to have seizures in silico. We found that the differences in excitatory currents obtained were exacerbated in patients who had a seizure within 72 h after the recording. Our findings indicate that further experimental work to investigate the links between sleep and seizure states may be a valuable line of enquiry for elucidating the pathways underlying seizure transitions.

Differences in brain activity outside of seizure states are well known to occur between patients with epilepsy and healthy controls.^6^, ^38^ Moreover, the distribution of interictal epileptiform activity is influenced by circadian rhythms,^39^ and the provocation of epileptiform discharges by sleep–wake transitions is already widely utilized to improve the diagnostic yield from visual EEG analysis in the clinical setting.^40^ Here, we propose that EEG during sleep can be used to uncover mechanistic differences in the brains of people with epilepsy. Future work should explore this hypothesis further by using mathematical models to generate predictions about the mechanisms underlying differences in brain activity observed in people with epilepsy across different states and timescales, and testing these predictions by quantifying treatment response.

### 4.1 Limitations

Limitations of our study include the small sample size and retrospective sample. Conversely, this meant that all seizures had been recorded as part of continuous study with the period of deep sleep used for analysis. The age range of subjects spanned the developmental period where SWA peaks then falls.^41^, ^42^ We minimized bias by ensuring that the control and patient groups were matched for age, although it is not possible to exclude that delays in the maturation of sleep homeostasis unrelated to seizure burden may have contributed to the observed differences. It was not possible to match patients and controls for full‐scale intelligence quotient or to control for variation in medication between patients in the seizure and nonseizure groups. These factors will be addressed in future studies using within‐patient comparisons.

### 4.2 Digital twinning to facilitate personalized antiseizure management

EEG remains the key investigation for the diagnosis and monitoring of epilepsy, with interpretation in the clinical setting by visual pattern recognition. However, recent advances in technology are improving biomarkers to help achieve the goals of seizure localization^38^ and forecasting.^43^ A further important question concerns the efficacy of treatment; a daily dilemma facing the clinician is how to select the best treatment for a specific patient, which can be conceptualized as an *n*‐of‐1 trial.

Building virtual simulations and integrating them with data to make inferences about clinical operations has been termed "digital twinning."^44^ Applying the modeling framework presented, the parameters optimized for each subject could serve as an in silico "twin" on which pharmacological, as well as nonpharmacological, interventions could be trialed virtually to aid clinical decision‐making. As an example, in this work we found the sensitivity of model dynamics was highest for changes in the excitatory conductance. This indicates that in this cohort the abnormal balance between excitation and inhibition, thought to underlie epilepsy, might be most effectively corrected by adjusting the excitation component. Therefore, to perturb slow‐wave sleep, the findings at the group level from the patients studied herein suggest that utilizing antiseizure medication that specifically modulates excitatory synapses, such as perampanel (a known AMPA receptor antagonist^35^, ^45^), could be most impactful and ultimately most beneficial for seizure control in children with focal lesional epilepsies. Further work will include assessing the use of the model on prospective data to determine whether it can predict the effects of interventions with known mechanisms. In this approach, within‐patient comparisons would counter bias due to variations in the maturity of SWA dynamics for age. This clinical validation will be essential in confirming the hypotheses derived from mathematical modeling.

### 4.3 Perspectives for guiding stimulation protocols

A further promising area of research concerns the manipulation of slow waves using targeted stimulation protocols. This approach has been implemented through various noninvasive methods, such as transcranial magnetic^46^ and acoustic^47^ stimulation. Deep brain stimulation could also be used with enhancement of SWA as a target outcome.^48^ Studies exploring stimulation to modulate activity have shown varied levels of success, from no systematic effect on the occurrence of spike–wave activity,^47^ to some significant improvements in patients with benign epilepsy.^49^ In the future, stimulation protocols could be tested by first implementing them in a mathematical model, such as the one discussed herein. For example, the parameters in deep brain stimulation, such as the stimulation frequency, amplitude, or duration, are currently dictated through a process of trial and error.^50^ It could be possible to optimize these parameters in a mathematical model, similar to the model used herein, by observing how the propensity to seizure changes in the model as a function of the stimulation parameters. The findings discussed in this study could help to explore ways of comparing resting data to seizure rhythms for quantifying this proximity. This could ultimately help dictate which combination of parameters is predicted to be most efficacious for alleviating seizures. Using a model to help systematize stimulation parameters would therefore be an interesting avenue for future research and could be crucial to help improve success rates for the nonpharmacological treatment of epilepsy.

## 5 CONCLUSIONS

By recovering parameters of a neural mass model from data, we demonstrate the feasibility of using mathematical models to elucidate mechanisms that may contribute to seizure burden in patients with epilepsy. Our results suggest that hyperexcitability underpins the discrepancies observed between children with epilepsy and healthy controls during slow‐wave sleep. Furthermore, by simulating seizures in the model, we provide evidence that the observed differences in this resting state may have a causative association with seizure propensity. This approach could generate new biomarkers for seizure susceptibility. Finally, by adjusting synaptic conductances in silico, we demonstrate a proof of concept for using mathematical models to hypothesize about the most efficacious interventions needed to rectify differences observed on the EEG, with the ultimate goal of improving patient outcomes.

## FUNDING INFORMATION

D.M.D. acknowledges the support of an Engineering and Physical Sciences Research Council DTP studentship (ref 2407565). S.Y.S.C. is funded by a St George's University of London Translational and Clinical Research Institute Senior Fellowship.

## CONFLICT OF INTEREST STATEMENT

None of the authors has any conflict of interest to disclose. We confirm that we have read the Journal's position on issues involved in ethical publication and affirm that this report is consistent with those guidelines.

## PATIENT CONSENT STATEMENT

Written informed consent was obtained from a parent of each participant.

## Supporting information


Data S1.

## Data Availability

The data that support the findings of this study are available on request from the corresponding author. The data are not publicly available due to privacy and ethical restrictions. The code used in this study has been made publicly available and is maintained as a GitHub repository (NeuralMassModellingrepository).
