# Supplementary material for: Neural mass modeling reveals that hyperexcitability underpins slow‐wave sleep changes in children with epilepsy
Source: Epilepsia. 2025 Feb 7;66(5):1652–64. doi: 10.1111/epi.18293 (PMC12097470; doi:10.1111/epi.18293)
Supplement: Supplementary file 1 — Data S1. [file EPI-66-1652-s001.pdf]

Supporting Information

Appendix S1: Neural Mass Model

Neural mass models (NMMs) represent the mean macroscopic behavior of populations of neurons. In this work, we employ the NMM developed by Weigenand et al [1]. This model consists of interacting excitatory and inhibitory neuronal populations and aims to recreate activity observed on the electroencephalogram (EEG) during non-rapid eye movement sleep.

The model dynamics are governed by differential equations that describe the change in activity (membrane potentials and synaptic dynamics) over time. Two main operators are used to generate the dynamics. The first transforms the average density of presynaptic input into a postsynaptic membrane potential. This is modeled by convoluting an impulse response function with the arriving input. The impulse response function used is given by the term $\alpha_j(t) = \gamma_j^2 t \exp(-\gamma_j t)$, for $j = e, i$ representing excitatory and inhibitory synapses, respectively. The second operator transforms the average membrane potential of a population into an outgoing firing rate. This is modeled by a nonlinear sigmoidal function, $S_j(V_j) = \frac{Q_j^{max}}{(1+exp(-\frac{\pi}{3}(V_j-\theta_j)/\sigma_j)}$, for $j = e, i$ representing the excitatory and inhibitory firing rate functions, respectively. The change in membrane potential of a population (excitatory or inhibitory) is then expressed as a summation of the current flow obtained from afferent neural populations. An example of an impulse response function and sigmoidal firing, along with a schematic of the model highlighting the interaction between the population of excitatory and inhibitory neurons, is given in Figure S1. In full, the model can be expressed as the following set of first and second-order differential equations:

$$\tau_e \frac{dV_e(t)}{dt} = -I_L^e(t) - s_{ee}(t)I_{ee}(t) - s_{ie}(t)I_{ie}(t) - \tau_e C_m^{-1} I_{KNa}(t), \tag{1}$$

$$\tau_i \frac{dV_i(t)}{dt} = -I_L^i(t) - s_{ei}(t)I_{ei}(t) - s_{ii(t)}I_{ii}(t), \tag{2}$$

$$\frac{d^2 s_{ee}(t)}{dt^2} = \gamma_e^2(N_{ee}S_e(V_e(t)) - s_{ee}(t) + \phi(t)) - 2\gamma_e \frac{ds_{ee}(t)}{dt}, \tag{3}$$

$$\frac{d^2 s_{ei}(t)}{dt^2} = \gamma_e^2(N_{ei}S_e(V_e(t)) - s_{ei}(t)) - 2\gamma_e \frac{ds_{ei}(t)}{dt}, \tag{4}$$

$$\frac{d^2 s_{ie}(t)}{dt^2} = \gamma_i^2(N_{ie}S_i(V_i(t)) - s_{ie}(t)) - 2\gamma_i \frac{ds_{ie}(t)}{dt}, \tag{5}$$

$$\frac{d^2 s_{ii}(t)}{dt^2} = \gamma_i^2(N_{ii}S_i(V_i(t)) - s_{ii}(t)) - 2\gamma_i \frac{ds_{ii}(t)}{dt}, \tag{6}$$

$$\tau_{Na} \frac{dNa(t)}{dt} = \alpha_{Na} S_e(V_e(t)) - R_{pump} \left(\frac{Na(t)^3}{Na(t)^3 + K_p^3} - \frac{Na_{eq}^3}{Na_{eq}^3 + K_p^3} \right). \tag{7}$$

The terms on the right-hand side of Equation 1 and Equation 2 are synaptic currents. These are derived as a consequence of Ohm's law (current equals the conductance multiplied by the voltage). Specifically, the term $-I_L^j = -g_L(V_j(t) - E_L^j)$ represents the passive leak current, with active currents given by, $-s_{jh}(t)I_{jh}(t) = -s_{jh}(t)g_j(V_h(t) - E_j)$ and $-I_{KNa}(t) = -g_{KNa}\frac{0.37}{1+(EC/Na(t))^{nH}}(V_e(t) - E_K)$, with $j = e, i$ and $h = e, i$. The voltage terms within the brackets indicate the difference between the membrane potential and the synaptic reversal potential. The currents therefore incorporate that the

postsynaptic current flow will be dependent on these values [1, 2]. The conductance terms consist of a conductance parameter g_j, for $j = L, e, i, KNa$ and, in the case of the active currents, are scaled by the synaptic activity, which is solved for in Equations 3-7. We note that in this model the KNa current was specifically introduced for modeling the sleeping cortex [1]. Furthermore, in the right-hand side of Equation 3, the term $\phi(t)$ acts as an external firing rate into the excitatory population of the model. This function incorporates activity from external unmodeled brain regions and is assumed to be a Brownian motion with variance scaled by ξ^2. This means Equations 1-7 are stochastic differential equations (SDEs).

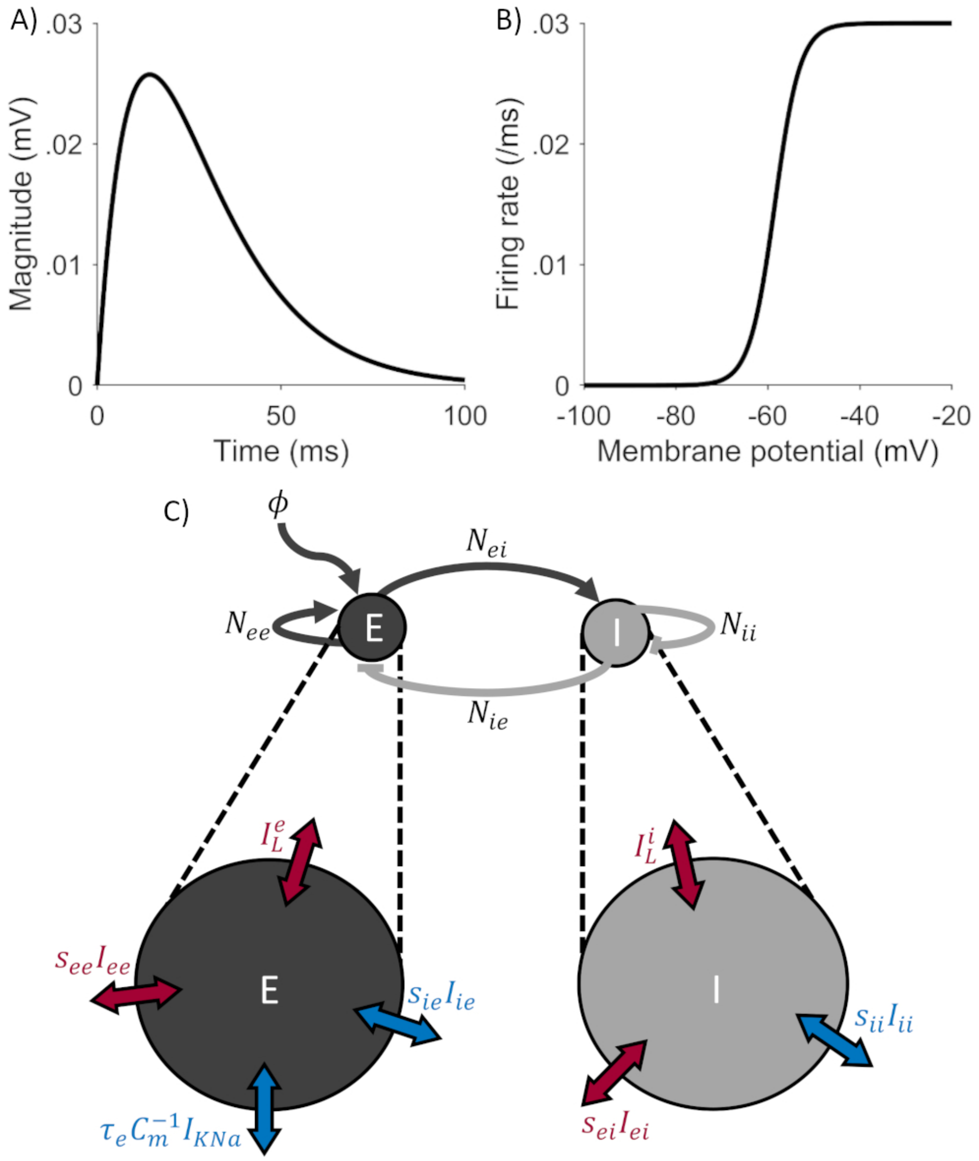

Figure S1: A) Example impulse response function used in the NMM. B) Example sigmoidal firing rate function used in the NMM. C) Schematic of the NMM, which consists of a population of interacting excitatory and inhibitory neurons. N_{ij} for $i, j = e, i$ indicate connections between populations. ϕ indicates the external input to the excitatory population. In each population, red labels indicate excitatory acting currents and blue labels indicate inhibitory acting currents (see model equations for further details). See Table S1 for a full list of model parameters.

In order to numerically solve the model, Equations 1–7 were converted into a set of eleven first-order SDEs. The Euler–Maruyama method was then used to numerically solve this set of SDEs, with zero

initial conditions. The model was simulated to match the length of the data (30 s for slow-wave activity, 2.5 s for spike-wave discharge) at a timestep of 0.2 ms to ensure accurate convergence. To account for transient dynamics, a further 5s was calculated at the start of the model simulation and then removed. The output of the model was taken to be the membrane potential of the excitatory population, $V_e(t)$. The model output was then downsampled to the same sampling frequency of the data (128 Hz), and the power spectra from model simulations were estimated in an identical way to the data (see subsection 2.2 EEG data acquisition and pre-processing in the main text). We highlight that no filtering of the model output was performed. When comparing experimental data and model output, both time series were z-scored before the power spectra were estimated. We note that the spike-wave discharge was simulated in the noise-free deterministic case (i.e. $\xi = 0$).

Table S1 provides a list of all the parameters in the model, along with a physiological interpretation, typically used values and parameter bounds. In general, these bounds were set to a 25% deviation from the typical values used (see Weigenand et al [1] and references therein). This is with the exception of the timescale parameters, in which their bounds were set to 100% of the typical values, and the synaptic reversal potentials for the excitatory and inhibitory populations, which were constructed to ensure that excitatory currents were depolarizing and inhibitory currents were hyperpolarizing (see Table S1). Moreover, Figure S2 shows the mean excitatory and inhibitory membrane potentials and synaptic currents on the excitatory population obtained from simulations of random parameter values within the bounds specified.

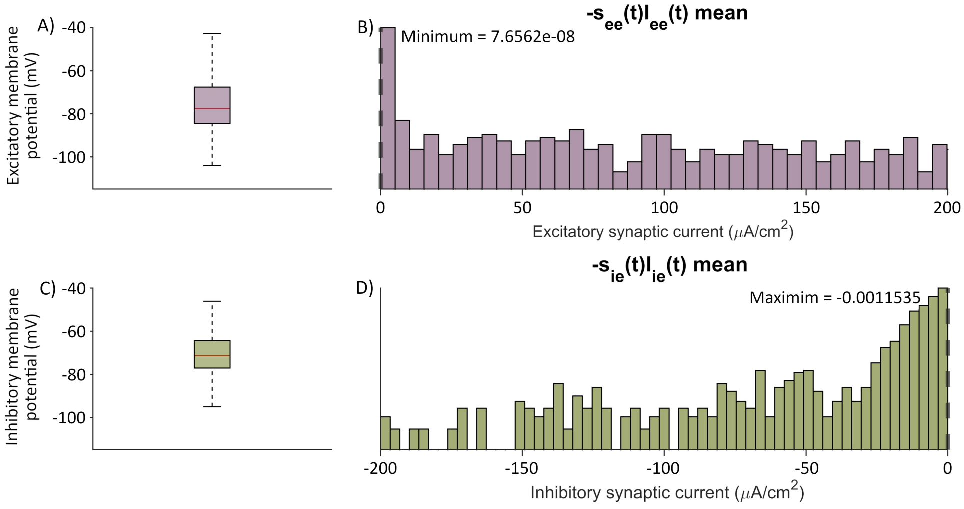

Figure S2: A) Mean excitatory membrane potential and B) mean excitatory synaptic currents obtained from simulations of 1000 random parameter sets within the bounds specified in Table S1. This range in membrane potentials is consistent with values stated from previous simulations with this model [1], and from simulations in the original incarnation of the model [2]. C) Mean inhibitory membrane potential and D) mean inhibitory synaptic currents obtained from simulations of 1000 random parameter sets within the bounds specified in Table S1. Excitatory synaptic currents are exclusively depolarizing within these parameter ranges (minimum current > 0) and inhibitory synaptic currents are exclusively hyperpolarizing (maximum current < 0).

Parameter	Interpretation	Typical Value	Bounds
N_{ee}, N_{ei}, N_{ie}, N_{ii}	Number of excitatory - excitatory/ excitatory - inhibitory/ inhibitory - excitatory/ inhibitory - inhibitory synaptic connections	120, 90, 72, 90	[90,150], [67.5,112.5], [54,90], [67.5,112.5]
γ_e, γ_i	Excitatory/ inhibitory postsynaptic potential rate constant	0.07 /ms, 0.0586 /ms	[0,0.14], [0,0.1172]
τ_e, τ_i	Passive excitatory/ inhibitory membrane time constant	30 ms , 30 ms	[22.5,37.5], [22.5,37.5]
Q_e^{max}, Q_i^{max}	Maximum mean firing rate of excitatory/ inhibitory population	0.03 /ms, 0.06 /ms	[0.0225,0.0375] , [0.045,0.075]
θ_e, θ_i	Excitatory/ inhibitory firing rate threshold	-58.5 mV, -58.5 mV	[-73.1250,-43.8750], [-73.1250,-43.8750]
σ_e, σ_i	Excitatory/ inhibitory firing rate standard deviation	5 mV, 6 mV	[3.75,6.25], [4.5,7.5]
E_e, E_i	Excitatory/ inhibitory mean synaptic reversal potential	0 mV, -70 mV	[-50,50], [-200,-100]
E_L^e, E_L^i	Excitatory/ inhibitory mean synaptic reversal potential associated with leak channels	-64 mV, -64 mV	[-80,-48], [-80,-48]
g_e, g_i, g_L	Excitatory/inhibitory/leak synaptic conductance	1 mS/cm^2, 1 mS/cm^2, 1 mS/cm^2	[0.75,1.25], [0.75,1.25], [0.75,1.25]
C_m	Specific membrane capacity	1 µF/cm^2	[0.75, 1.25]
R_{pump}	Sodium pump capacity	0.09 mMm/s	[0.0675,0.1125],
τ_{Na}	Sodium time constant	1.3 ms	[0.975,1.625]
α_{Na}	Sodium influx	2 mMm/s	[1.5,2.5]
Na_{eq}	Sodium resting state	9.5 mM	[7.125,11.875]
g_{KNa}	Synaptic conductance associated with sodium-potassium pump	3 mS/cm^2	[2.25,3.75]
E_k	Mean synaptic reversal potential associated with sodium-potassium pump	-100 mV	[-125,-75]
EC	Sodium for half activation	38.7	[29.025,48.375]
n_H	Hill coefficient	3.5	[2.625,4.375]
K_p	Pump constant	15	[11.25,18.75]
ξ	Standard deviation of noise perturbation	-	[3.75,6.25]

Table S1: Model parameters with interpretation, a typically chosen value [1] and parameter bounds.

Appendix S2: Multiobjective Optimization

In this study, we used a multiobjective evolutionary algorithm to recover parameters from EEG data. In particular, we used the Non-dominated Sorting Genetic Algorithm-II (NSGA-II) [3]. A central reason for using NSGA-II is because of the complex bifurcation structure that is known to exist in an NMM. Evolutionary algorithms, such as NSGA-II, are stochastic search methods inspired by natural selection. They aim to solve an optimization problem by combining a number of individuals into a population, where each individual is a set of parameters that produce a model simulation. The model simulation is then compared to data, and based on preassigned objectives, the 'fitness' of the given individual is calculated. A group of individuals forms a population at a given generation. The algorithm then iterates through generations by applying various mutation and crossover operators on individuals that present better fitness values, in an aim to find the best individuals that can minimize the fitness. The algorithm is terminated when the number of iterations is high enough to ensure the fitness values obtained are minimized. Due to nonidentifiable parameters, when using these algorithms for parameter estimation in neural mass models, this process is often repeated to obtain a distribution of parameters that generate a model simulation with a good approximation (or fitness) of the data. We refer the reader to previous work for further information on the use of evolutionary algorithms in parameter estimation of neural mass models in simulating data from patients with epilepsy [4, 5, 6].

In general, multiobjective optimization aims to minimize **F(x)** $= (f_1(\mathbf{x}), f_2(\mathbf{x}), ..., f_d(\mathbf{x}))$, given a set of constraints on the inputs **x** $= (x_1, x_2, ..., x_g)$. **F(x)** forms the d-dimensional objective space and **x** forms the g-dimensional decision space. In this study, the decision space (or model parameter inputs) is bounded by the constraints on parameters (as defined in Table S1). Following our previous work [6], we define two objectives (i.e. $d = 2$) to describe the difference between the model simulation and the data. The first objective is the sum of squared error between the normalized power from the model simulation and the normalized power from the data (after the data was filtered by a 4th order Butterworth filter between .3 Hz and 10 Hz). This is defined by,

$$Obj_1 = \sum_{\omega_1} (\mu^{\omega_1}_{data} - \mu^{\omega_1}_{model})^2, \tag{8}$$

where $\mu^{\omega_1}_{data}$ gives the data normalized power at frequency ω_1 and $\mu^{\omega_1}_{model}$ gives the model normalized power at frequency ω_1. In the interest of modeling slow-wave sleep, ω_1 was calculated across .6 Hz to 10 Hz, with a resolution of .025 Hz.

The second objective used is based on the horizontal visibility graph (HVG) algorithm, which provides a mapping from a time series to an undirected network [7]. Here, each time point becomes a node in the network and an edge is drawn between two nodes in the network if it is possible to trace a horizontal line between the two data points, without intersecting an intermediate point. Formally, for a time series $\{a_i\}_{i=1,....,N}$ of N data points, the HVG algorithm sets nodes $V = \{1, ..., N\}$ and edges between nodes i and j iff $a_i, a_j > a_k \; \forall \; i < k < j$. In this study, we used the extended version of this algorithm known as the weighted HVG, whereby if there exists an edge between two nodes, then that edge is weighted by the difference in amplitude between the time points (latter time point minus former time point). Retaining properties of the weighted HVG in NMM simulations have previously been shown to be useful in terms of refining the plausible dynamics [6]. We use the two-sample Kolmogorov-Smirnov test statistic to generate the objective for the difference between the weighted HVG from the model simulation and the data. In particular, for $F : \mathbb{R} \to [0, 1]$ and $G : \mathbb{R} \to [0, 1]$ representing the weighted HVG sum of node weights

cumulative distribution function of the data and model simulation respectively, and $y \in \mathbb{R}$, the second objective is defined as,

$$Obj_2 = \sup_y \mid F(y) - G(y) \mid . \tag{9}$$

Qualitatively different dynamics can yield the same normalized power, for example, due to differences in amplitude or phase distributions [8]. Following our previous work [6], incorporating properties of the weighted HVG as an objective enabled the types of model simulations to be refined and improved model identifiability. Figure S3 shows an example simulation recovered from a single objective (difference in normalized power) and a two-objective (difference in normalized power and difference in weighted HVG distribution) optimization. The single objective approach produces simulations that accurately reproduce the normalized power of the data, but that produce spurious waveforms in the time domain. This is reflected by a different weighted HVG degree distribution compared to the data. Conversely, the two-objective approach still produces a simulation with good agreement to the normalized power, but also a simulation that better resembles the data in the time domain, as evidenced by a good agreement in the weighted HVG distribution. We refer the reader to Schindler et al [9] for further information on the use of the weighted HVG for refining simulated nonlinear brain rhythms in epilepsy.

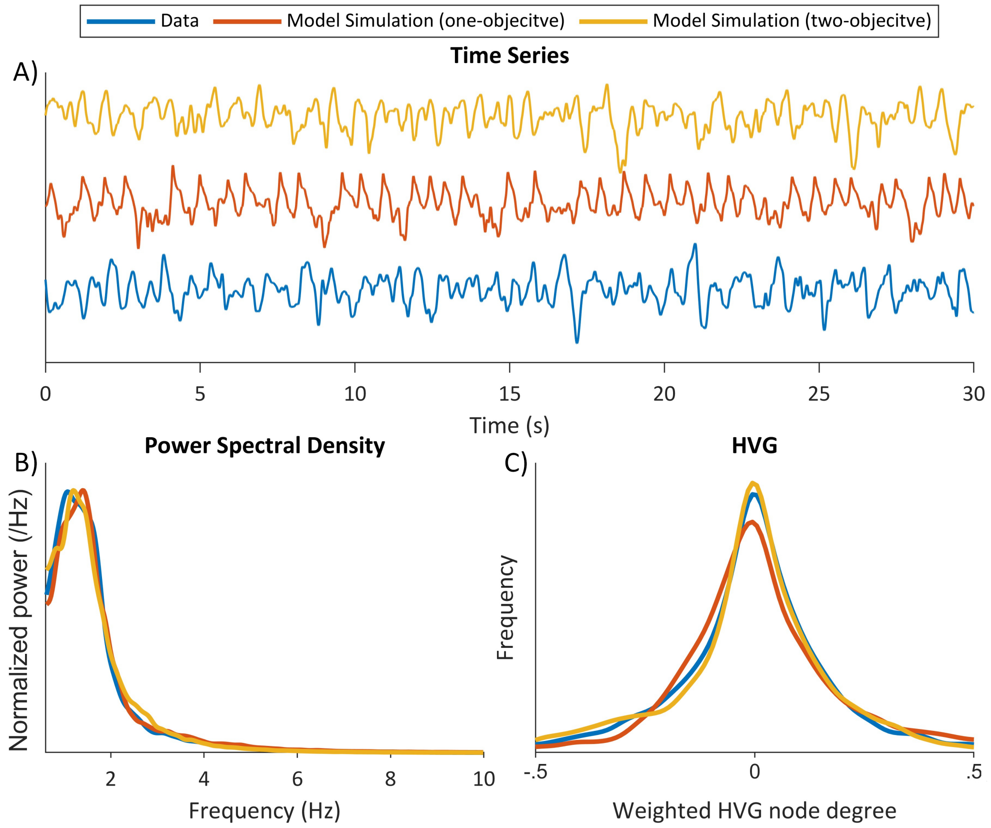

Figure S3: A) Example time series, B) normalized power and C) weighted HVG distribution for an example model simulation after optimizing to the data using the one-objective and two-objective approaches. In this example, the one-objective approach produces a simulation that, in the time series, does not appear to recapitulate the data (the simulation appears to have periodic spiking). Incorporating the weighted HVG for the second objective helps to refine the type of model dynamics recovered.

Due to the stochasticity of the model simulation, and to ensure accurate objective calculations, we simulate the model and calculate each objective function twenty times and then assign the mean value obtained from these repeats as the objective for that parameter set. We note that all parameters were varied in the optimization, and a fixed population size of 500 and a generation number of 50 were used for accurate convergence.

Appendix S3: Epilepsy Characteristics

Table S2 gives details on the individual patient characteristics.

Patient ID	Age	Age of epilepsy onset	Duration of epilepsy	Sex	Etiology	Seizure focus	Seizure frequency	Seizure(s) during admission	Antiseizure medication(s)
1	9.8	4.8	5	F		Occipital	Weekly	No	Levetiracetam
2	15.5	6	9.5	M	Focal Cortical Dysplasia	Temporal	Monthly	No	Lamotrigine
3	15.3	13.5	1.8	M	Glioneuronal tumour	Temporal	Monthly	No	Lamotrigine, Sodium valproate
4	9.2	2	7.2	F	MRI negative	Undetermined	Weekly	Yes	Clobazam
5	10.4	3	7.4	M	MRI negative	Frontal	Daily	Yes	Carbamazepine
6	12.6	2	10.6	M	Ganglioglioma	Temporal	Weekly	Yes	Levetiracetam
7	6.6	2	4.6	F	Focal Cortical Dysplasia	Fronto-temporal	Monthly	No	Carbamazepine
8	14.8	12	2.8	F		Parietal	Weekly	No	None
9	12.6	4	8.6	M		Fronto-temporal	Daily	Yes	Lamotrigine
10	14.8	8	6.8	M	Focal Cortical Dysplasia	Fronto-temporal	Weekly	Yes	Levetiracetam, Sodium valproate, Oxcarbazepine
11	11.1	2	9.1	M	MRI negative	Fronto-temporal	Weekly	Yes	Carbamazepine, Topiramate
12	7.8	2	5.8	M		Undetermined	$<$1 per month	No	Levetiracetam, Topiramate, Clobazam
13	11.3	8	3.3	M	MRI negative	Undetermined	$<$1 per month	No	Levetiracetam, Sodium valproate
14	12.8	8	4.8	M	MRI negative	Frontal	Daily	Yes	Lacosamide
15	7	0.9	6.1	M	Focal Cortical Dysplasia	Frontal	$<$1 per month	No	Levetiracetam, Sodium valproate

Table S2: Patient demographics.

Appendix S4: Results from comparing control and patient models

To calculate the statistical significance of differences between distributions of parameters recovered from controls and patients, in Figure S6, we generated surrogate Jensen-Shannon divergences by randomly permuting labels from the combined distributions. We repeated this process 1000 times via bootstrap sampling. We compared the surrogate Jensen-Shannon divergences to the Jensen-Shannon divergence obtained from control vs patient distributions to obtain a p-value. This p-value was corrected for multiple comparisons via Bonferroni correction (i.e. corrected by multiplying by 32, due to there being 32 parameters, *$p<0.05$ after this correction). As described in the manuscript, pairwise comparisons were computed using a Mann-Whitney U test in Figures S7 and S8.

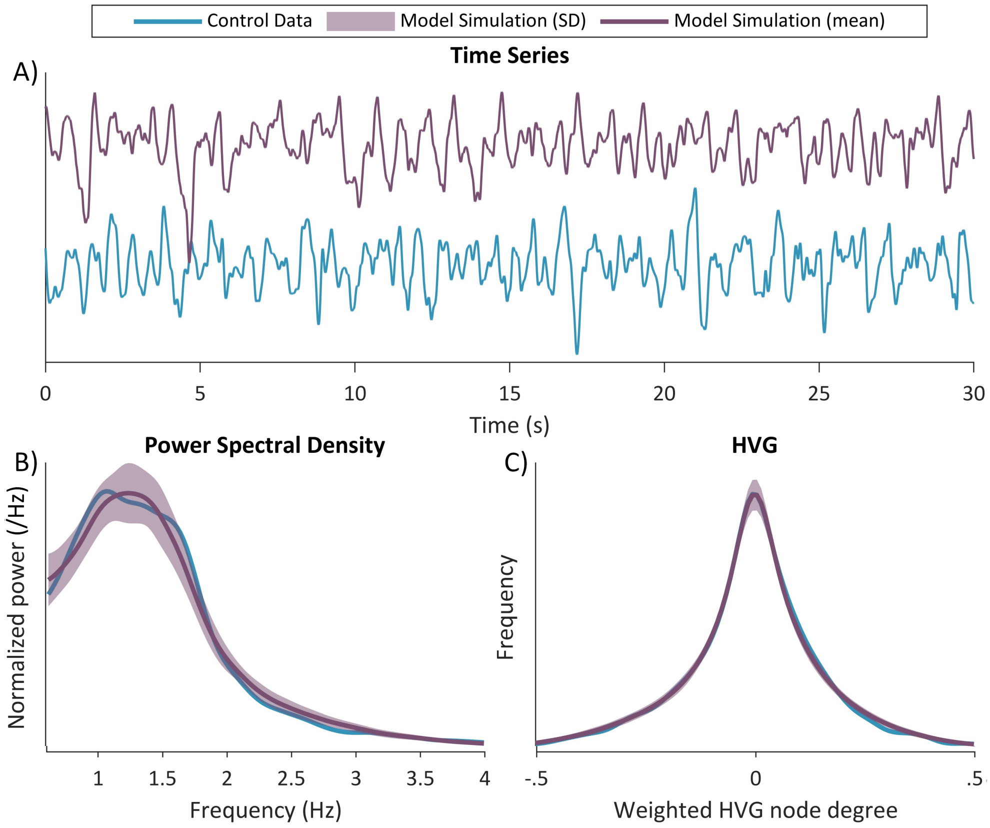

Figure S4: A) Example time series, B) normalized power and C) weighted HVG distribution for a control data subject and model output, after optimizing the model to data. The example shows the model dynamics closely replicate properties of the EEG data.

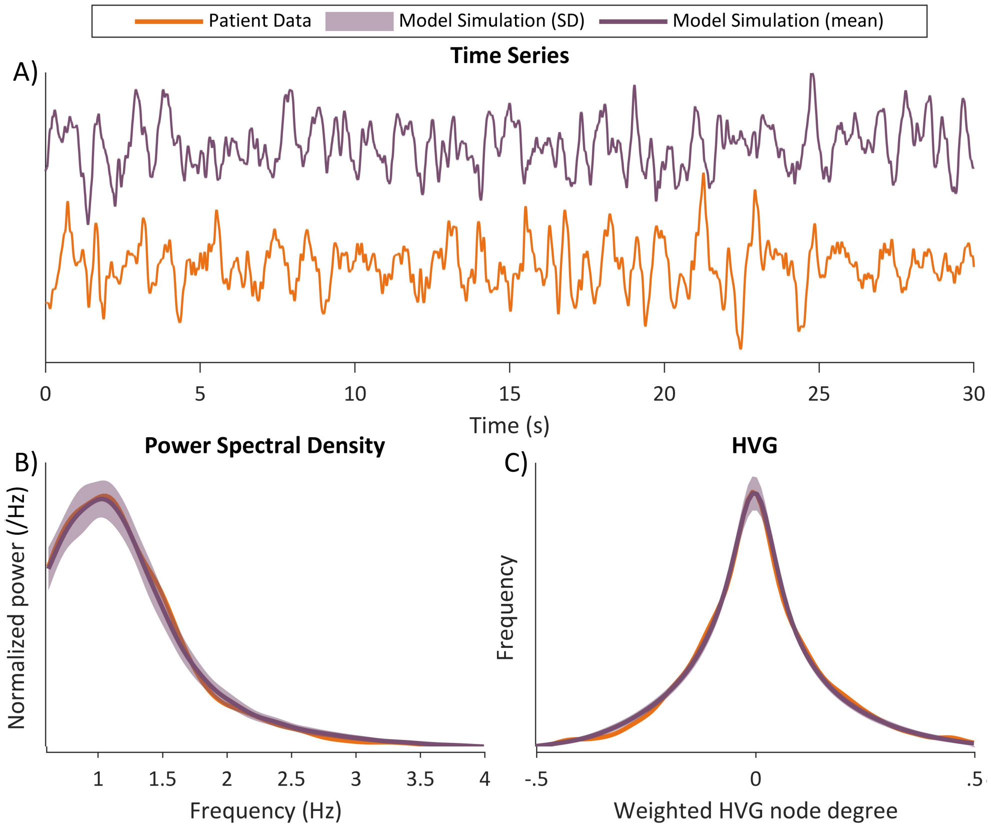

Figure S5: A) Example time series, B) normalized power and C) weighted HVG distribution for a patient data subject and model output, after optimizing the model to data. The example shows the model dynamics closely replicate properties of the EEG data.

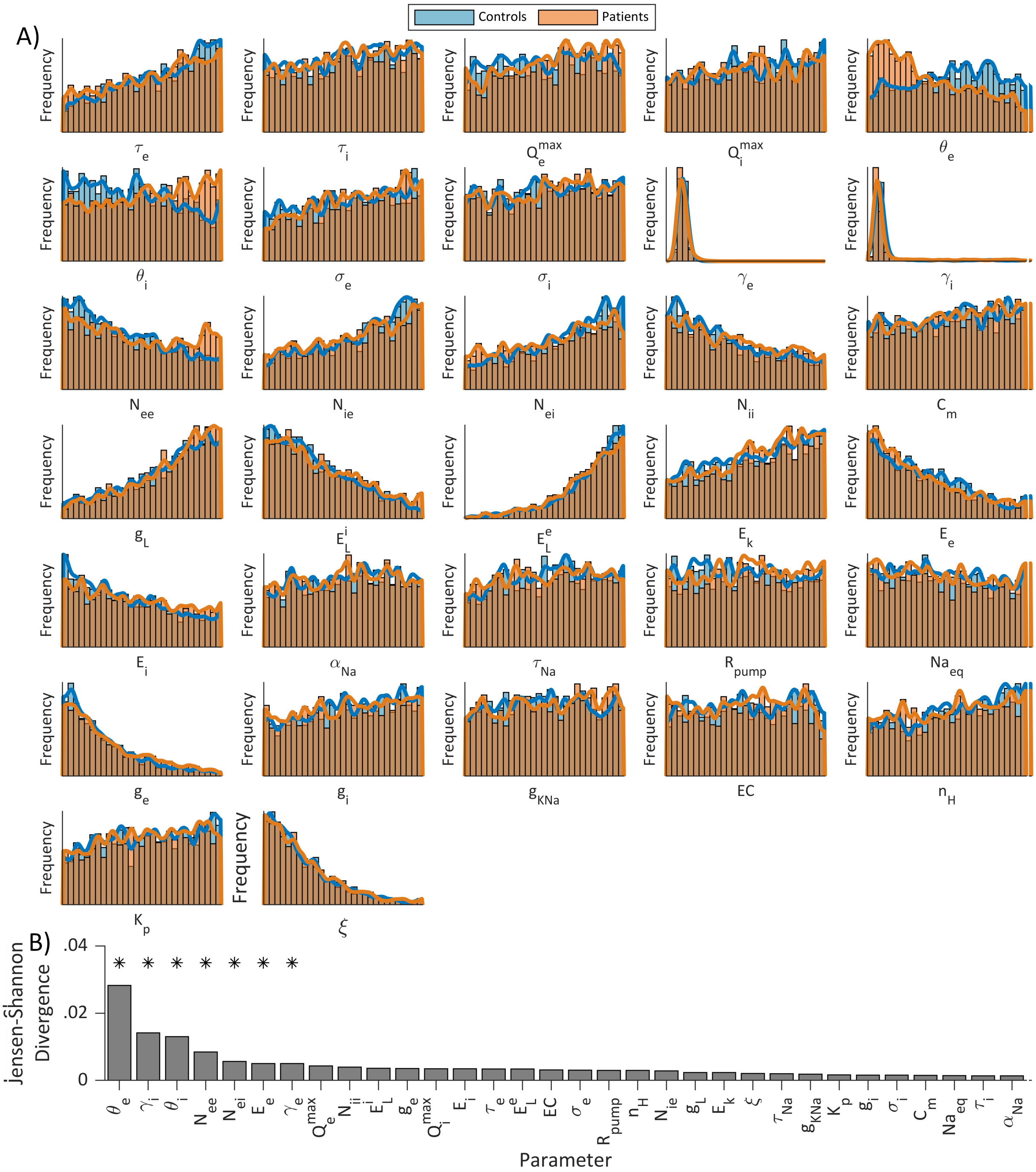

Figure S6: Parameter distributions recovered from fitting to all control and patient subjects. A) Univariate parameter distributions. B) Jensen-Shannon Divergence between controls and patients for each parameter. * Gives parameters with significant differences between controls and patients after Bonferroni correction.

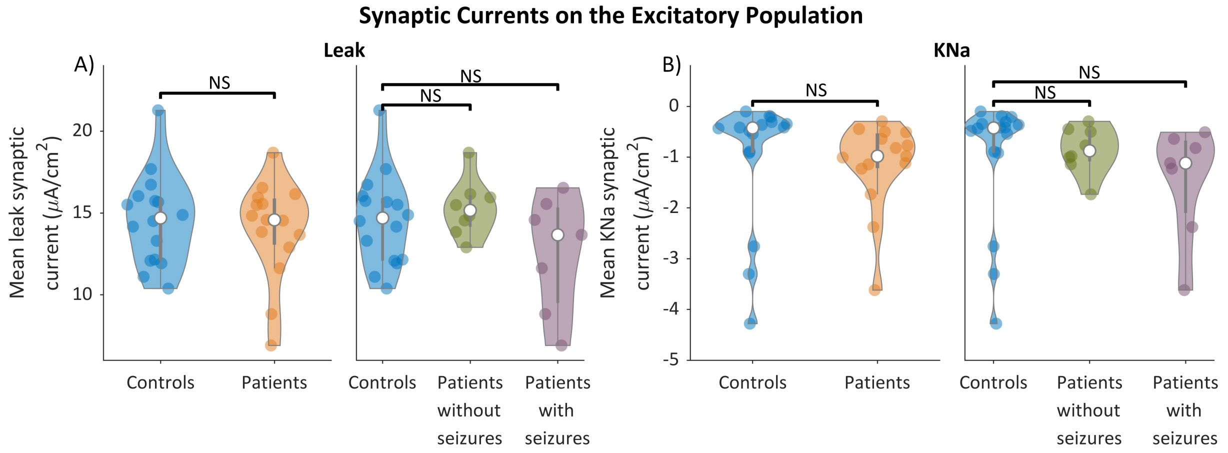

Figure S7: A) Leak and B) KNa synaptic currents on the excitatory neuronal population, obtained from model simulations. In each case, each point on a violin plot gives the mean value of a subject. No significant differences were found (NS = not significant) after Bonferroni correction.

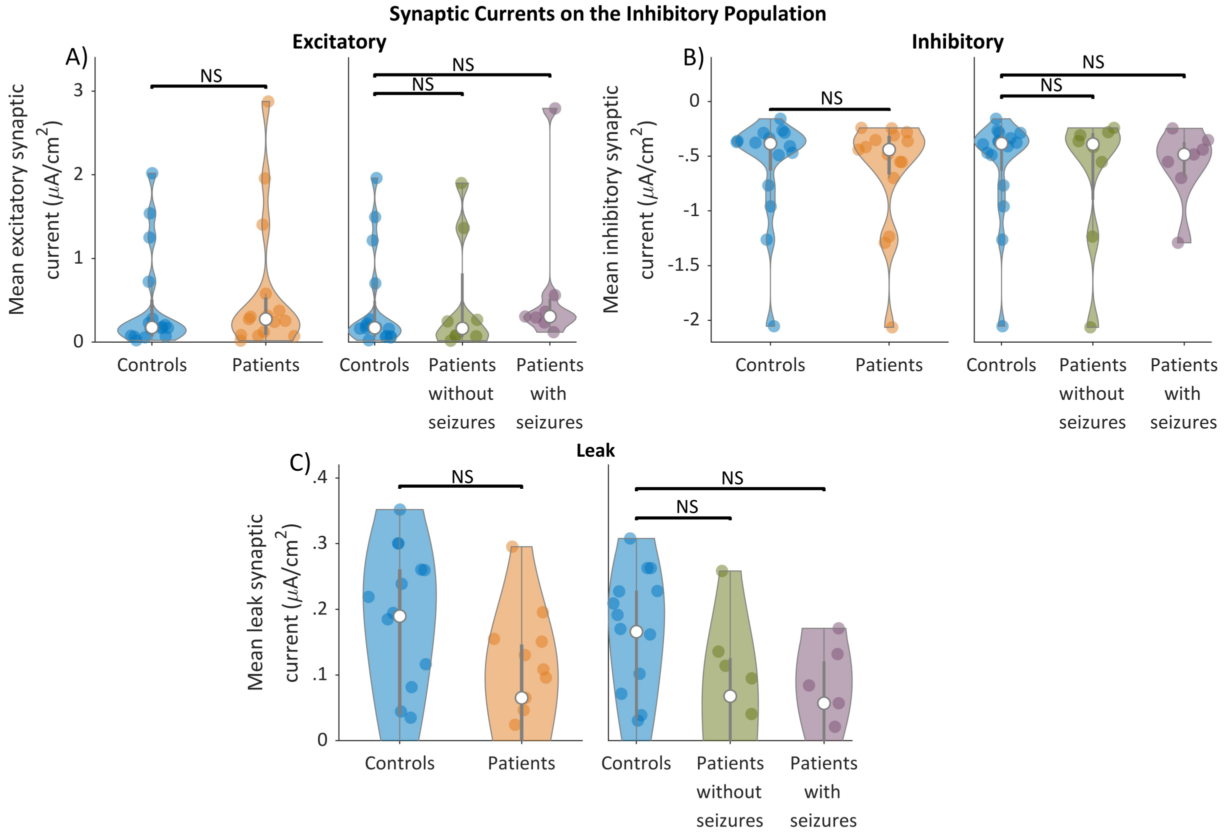

Figure S8: A) Excitatory, B) inhibitory and C) leak synaptic currents on the inhibitory neuronal population, obtained from model simulations. In each case, each point on a violin plot gives the mean value of a subject. No significant differences were found (NS = not significant) after Bonferroni correction.

References

[1] Weigenand A, Schellenberger Costa M, Ngo HV, Claussen JC, Martinetz T. Characterization of K-complexes and slow wave activity in a neural mass model. PLoS Comput Biol. 2014;**10**(11):e1003923.

[2] Liley DT, J CP, Dafilis MP. A spatially continuous mean field theory of electrocortical activity. Netw Comput Neural Syst. 2002;**13**(1):67–113.

[3] Deb K, Pratap A, Agarwal S, Meyarivan T. A fast and elitist multiobjective genetic algorithm: NSGA-II. IEEE Trans Evol Comput. 2002;**6**(2):182–197.

[4] Wendling F, Hernandez A, Bellanger JJ, Chauvel P, Bartolomei F. Interictal to ictal transition in human temporal lobe epilepsy: insights from a computational model of intracerebral EEG. J Clin Neurophysiol. 2005;**22**(5):343–356.

[5] Nevado-Holgado AJ, Marten F, Richardson MP, Terry JR. Characterising the dynamics of EEG waveforms as the path through parameter space of a neural mass model: Application to epilepsy seizure evolution. NeuroImage. 2012;**59**(3):2374–2392.

[6] Dunstan DM, Richardson MP, Abela E, Akman OE, Goodfellow M. Global nonlinear approach for mapping parameters of neural mass models. PLoS Comput Biol. 2023;**19**(3):e1010985.

[7] Luque B, Lacasa L, Ballesteros F, Luque J. Horizontal visibility graphs: Exact results for random time series. Phys Rev E. 2009;**80**(4):046103.

[8] Stam C, Pijn J, Suffczynski P, Lopes da Silva F. Dynamics of the human alpha rhythm: evidence for non-linearity? Clin Neurophysiol. 1999;**110**(10):1801–1813.

[9] Schindler K, Rummel C, Andrzejak RG, Goodfellow M, Zubler F, Abela E, et al. Ictal time-irreversible intracranial EEG signals as markers of the epileptogenic zone. Clin Neurophysiol. 2016;**127**(9):3051–3058.
